# Recent Progress of Gold-Based Nanostructures towards Future Emblem of Photo-Triggered Cancer Theranostics: A Special Focus on Combinatorial Phototherapies

**DOI:** 10.3390/pharmaceutics15020433

**Published:** 2023-01-28

**Authors:** Rajkumar Sekar, Nagaraj Basavegowda, Jesse Joel Thathapudi, Medidi Raja Sekhar, Parinita Joshi, Prathap Somu, Kwang-Hyun Baek

**Affiliations:** 1 Department of Chemistry, Karpaga Vinayaga College of Engineering and Technology, GST Road, Chinna Kolambakkam, Chengalpattu 603308, India; 2 Department of Biotechnology, Yeungnam University, Gyeongsan 38541, Republic of Korea; 3 Department of Biotechnology, School of Agriculture and Biosciences, Karunya Institute of Technology and Sciences (Deemed-to-be University), Karunya Nagar, Coimbatore 641114, India; 4 Department of Chemistry, College of Natural Sciences, Kebri Dehar University, Korahe Zone, Somali Region, Kebri Dehar 3060, Ethiopia; 5 SDM College of Medical Science and Hospital, Manjushree Nagar, Sattur, Dharwad 580009, India; 6 Department of Bioengineering, Institute of Biotechnology, Saveetha School of Engineering, SIMATS, Chennai 600124, India

**Keywords:** cancer therapy, gold nanostructures, synergetic therapies, nanomaterials, biomedical applications

## Abstract

Cancer is one of the most dangerous health problems in the millennium and it is the third foremost human cause of death in the universe. Traditional cancer treatments face several disadvantages and cannot often afford adequate outcomes. It has been exhibited that the outcome of several therapies can be improved when associated with nanostructures. In addition, a modern tendency is being developed in cancer therapy to convert single-modal into multi-modal therapies with the help of existing various nanostructures. Among them, gold is the most successful nanostructure for biomedical applications due to its flexibility in preparation, stabilization, surface modifications, less cytotoxicity, and ease of bio-detection. In the past few decades, gold-based nanomaterials rule cancer treatment applications, currently, gold nanostructures were the leading nanomaterials for synergetic cancer therapies. In this review article, the synthesis, stabilization, and optical properties of gold nanostructures have been discussed. Then, the surface modifications and targeting mechanisms of gold nanomaterials will be described. Recent signs of progress in the application of gold nanomaterials for synergetic cancer therapies such as photodynamic and photo-thermal therapies in combination with other common interventions such as radiotherapy, chemotherapy, and will be reviewed. Also, a summary of the pharmacokinetics of gold nanostructures will be delivered. Finally, the challenges and outlooks of the gold nanostructures in the clinics for applications in cancer treatments are debated.

## 1. Introduction

Cancer is a leading public health issue and the second primary reason for death at the global level. In 2020, nearly 10 million deaths were reasoned due to cancer as reported by the World Health Organization. Incredibly, about six out of one death globally is thought to be because of cancer [1]. The rising awareness of the molecular and cellular facts that reason cancer has permitted the advancement of novel approaches to handle this disease [2]. Still, the most general and traditional therapy surgery for clearing the tumor tissue is tailed by chemo/radiotherapy, either single or multi-modal, which is commonly based on the category and spreading of cancer. There is no united method to handle all categories of cancer, however, single-mode treatment not exhibiting a cent percentage efficiency for each case [3,4]. The conventional interventions for cancer have exhibited certain boundaries because of the absence of targeting ability toward the malignant consequently it rises several side effects. Moreover, these conventional treatments are incapable to eliminate the tumors from the body. These traditional approaches can also generate strong specific pressure aids to develop resistance against the therapy [5,6,7]. The golden standard method depends on the surgical clearances of cancer-affected parts, which is a promising way for the initial stage of the disease. Though, imperfect resection may again seed the malignant cells at the infected region, which then are prone to cancer regeneration [8]. Chemotherapy utilizes organic molecules-based drugs to control and abolish the cancer cell multiplications, whose restricted efficiency, heavy cytotoxicity, and multi-drug resistance of cancer cells have induced the advancement of novel organic/inorganic compounds [9,10,11]. Commonly, chemotherapy is jointly applied with surgery, radiotherapy, and immunotherapy [7]. Radiotherapy utilizes high-energy ionizing radiation to destroy the tumor sites. Usually, radiotherapy is a supportive approach to chemotherapy to enhance the ablation of tumor sites [8,12,13]. Based on these limitations of these traditional therapies have led to the development of modern approaches, from synergetic therapies that depend on regular anticancer drugs to radial novel strategies that make use of advanced equipment [14].

Among these modern treatments, phototherapy has gained enormous attention and emerged as an alternative to traditional cancer therapies. Phototherapy abolishes cancer cells by selectively triggering photochemical and/or photothermal processes. The purpose of phototherapy is to abolish cancer cells by generating reactive oxygen species (ROS) or heat using photoactive agents. Phototherapy can be divided into two types according to their different mechanisms of action: photodynamic therapy (PDT) and photothermal therapy (PTT) [15]. Nanoparticle-based phototherapies have encouraged novel strategies that involve the specific targeting ability of biomolecules to the tumor microenvironments, the prospect of applying novel forms to combine various treatments such as photodynamic therapy and photothermal ability, in combination or single mode. Nanotechnology-based cancer therapies may provide various exciting opportunities for the advancement of novel approaches for imaging and therapy as well as enhances the efficiency of currently existing treatments [5,7,8,15,16].

In recent years, many multifunctional nanocarriers have been reported applying numerous kinds of organic/inorganic nanoparticles in which different components may be joined into one nanoplatforms for synergetic imaging and therapy of cancer. Commonly, organic-based nanocarriers comprise dendrimers, polymers, and liposomes nanoparticles; whereas inorganic-based nanocarriers prominently consist of magnetic, plasmonic, mesoporous silica nanoparticles, quantum dots, and carbon-mediated nanomaterials, among others [17,18]. Heterocyclic structure-based organic nanoparticles have several for clinical photodynamic treatments, such as quick chemical functionalization, minimum cytotoxicity, hemocompatibility, and biodegradability in physiological conditions [18,19]. Though organic nanoparticles are only triggered by ultraviolet-visible sources and in some specific cases include near-infrared radiation also, which may restrict direct application and specific targeting ability to therapy [17]. On the other hand, inorganic-based nanoparticles particularly noble metals nanoparticles (Au, Ag, Pt, Pd) usually have good near-infrared (NIR) radiation absorption, which is more promising for deep penetration on phototherapies. For instance, Zhu et al., (2016) have reported the excellent photothermal effect of poly(diallyl dimethylammonium chloride)-coated porous Pt nanoparticles where more than 70% of cancer cells are photothermally ablated after 808 nm laser irradiation for 3 min at 8.4 W cm^−2^ [19]. Further, NIR dye (CyOH)–coated silver nanoparticle/carbon dot nanocomposite exhibited excellent photodynamic potential by the production of a high singlet oxygen yield upon 660 nm laser irradiation leading to mitochondrial accumulation of nanophotosensitizer, superior tissue penetration, and enhanced significant antitumor effect [20]. These inorganic-based nanocarriers are usually non-biodegradable and accumulate on various healthy parts of the body, consequently inducing inflammations [19]. In recent years, several varieties of inorganic nanoparticles have been reported among them gold nanoparticles owing to their special physicochemical characteristics have been extensively applied for the advancement of novel therapeutic strategies [21]. Gold nanoparticles (GNPs) are easy to prepare on a large scale in an aqueous medium, which can quickly surface modified with a variety of therapeutic agents, encouraging them to optimal nanocarriers for synergetic bio-imaging and therapeutic applications. There are many opportunities for bioconjugation either through quick chemical bonding with thiol groups or dual functionalization or complex chemical conjugations [5,22,23,24]. However, the approach applied for the surface modifications of gold nanoparticles (GNPs) is the precise and quantitative measurement of the functionalized ligands and is essential to be considered as the major factor for succeeding uses. The localized surface plasmon resonances of GNPs are of outstanding merit toward the advanced photo-stimulated treatments, photodynamic therapy (PDT), and photothermal therapy (PTT) which offer more specific targeting ability and higher therapeutic efficiency [25,26]. The light energy transported by photons resources may be transformed into thermal energy or acoustic waves [15]. Even though the successful reports from approaches in the combat against cancer, the combination of GNPs mediated phototherapies associated with traditional therapies offers more therapeutic efficiency. Over the three last decades, PDT has been investigated pre-clinically for various cancer types, particularly for specific cancers for instance oropharyngeal, esophageal, and cutaneous [7]. Because of the minimum penetration power of ordinary ultraviolet-visible resources into the organ, PDT has not been applied for the therapies of various cancers that are originating inside the vital organs to date. The issue of low penetration is still a more challenging one and needs to be overwhelmed. Moreover, to enhance PDT for cancer, further standardization and the advancement of therapy approaches using PDT combined with already existing cancer therapies are required. Herein, the review focus on the applications of GNPs in light-triggered phototherapies in cancer. The combination of various other therapies with phototherapies based on gold nanostructure is the special focus of this review, together with the current challenges pebbledash the pre-clinical translation of these GNPs phototherapies. Additionally, chemotherapy, phototherapies, and immunotherapy, among others, are outstanding synergetic therapies for PDT. Moreover, the optical properties, side effects, the mechanisms of PDT based on GNPs are also highlighted. Finally, this review provides a better understanding of PDT combination approaches using gold nanostructure as the core and also how gold-based nanomedicine can aid to improve the therapeutic efficacy of these combinations.

## 2. Light-Based Cancer Therapies

Light develops physiological changes in cells and stimulates an endogenous biological chemical reaction either directly or indirectly [8,24]. The light-based biochemical activity can be extensively classified into those relying on the applications of photoconversion efficiency. In PDT, organic/inorganic nanoparticles can absorb light, this will then stimulate the chemical changes leading to the therapeutic outcome. While in PTT, the absorbed light by inorganic nanoparticles will stimulate the transformation of irradiating laser into localized thermal energy [8,19]. Other approaches utilize the capacity of GNPs to develop high energetic radiation (radiotherapy) to focus therapy on the cancer region. The potential merits of phototherapies are minimal cytotoxicity, invasive, and specific tumor ability [17]. Commonly, lasers are applied as radiation sources for photo-based cancer theranostics which can produce a monochromatic light that can be allowed via an optical fiber and attainted the target region directly [25]. The wavelength of the laser irradiation should depend on the capability to absorb the light by photosensitizers (PS), the location of the tumor, size, and various other factors to standardize the activation of the PS [27,28]. For effective clinical practice, phototherapies are strongly dependent on wavelength, exposure duration, penetration power, mode of delivery, and total dose of the light [29]. From 600 to 800 nm is the common wavelength range applied for phototherapies named a therapeutic window, within this wavelength, the energy of radiation can excite the PS permitting efficient tissue penetration but restricting the absorption of light by other cellular parts (cytochromes) [28]. PDT consists of three potential components as Oxygen, PS, and light source for cancer therapy [30]. One major advantage of PDT, it can proceed in the repetitive mode without generating immune/myelosuppressive effects and is also administered even after traditional therapies. The standard PS ligand should be a single pure molecule that permits quality assurance research with resonance stability. The PS can administer via topical or intravenous injections. However, the alteration in biodistribution over a long duration gets affected; the alternative path to regulate the impact of PDT is the duration of light exposure.

The laser absorption, the sensitizer, is transformed from a single state (short-lived) to a triplet state (long-lived). This triple state responds in two types such as (i) triple state reacts directly to the cell membrane and transfers the molecules into free radicals. When these free radicals react with oxygen molecules to form the oxygenated products (type I). (ii) Another way, the triple state can transfer its energy to the oxygen molecules and transform the singlet oxygen into a reactive oxygen species (type II). However, commonly all PDT drugs are oxygen-dependent, this therapy does not work in the anoxia region of the infected organ. Several reported in vivo investigations showed that holding tissue hypoxia reduces the PDT effects [31]. Types I and II are generated through specific mechanisms, which are occurred mutually and depend on the sensitizer type, substrate, and the number of oxygen molecules that exists in the tumor microenvironment. PDT-based tumor ablation involves three major pathways. First, PDT produced reactive oxygen species that directly attack the cancer cells and injured the tumor-combined vasculature, consequently removing the tumors. The three pathways can also influence each other [32]. The major limitations of PDT, are only applicable in the cancer region where laser radiation penetration is accessible, thus this PDT is chiefly adaptable for the lining organs, considering that laser cannot pass throughout the body tissues. PDT cannot be applied to clear large tumors and cancers that blow out to the majority of sites. The types of PSs applied in PDT circulate in the body for a longer time, which makes the patient more sensitive to laser radiation for a short while. Thus, attention should be considered after the administration of the PSs inside the body [33,34,35,36,37].

Near-infrared radiation has been widely applied to produce thermal spots and serve the resolution. Compared to other techniques, PTT has a great attraction to offer excellent therapeutic value because of its low invasiveness, enhanced therapeutic efficiency, low side effects, no need for long-lasting therapy, and fast recovery [38]. A photothermal agent can be allowed in the tumor microenvironment region and absorb the NIR laser lights to generate kinetic energy that releases thermal energy on the cancer cells and lead to cell death [39]. The permitted capacity of NIR absorption on healthy organs in the wavelength between 650–1350 nm develops crucial interpenetration in the patient’s body and destroys the tumors [40]. Additionally, this technique develops thermal waves to generate trouble in the cell membrane of the adjacent cancer cells [41]. Hyperthermia via absorption of NIR laser develops some problems such as short inter-penetrating power and strong absorption which leads to developing injury on normal cells and reducing the therapeutic efficiency. In the case of surface plasma resonances holding nanostructures, showed the electrons on the conduction band were localized on the surface of the nanomaterials upon NIR applications, and subsequently the localization attaints the target at the resonant frequency to develop resonances, thereafter absorbed NIR transforming into thermal waves. Therefore, it is probable to control NPS via local intravenous injection and consequently, exciting them in the NIR open windows I and II. Additionally, the nanomaterials incline to deposits in the spleen, kidney, and liver inducing injury irreversibly [42,43]. Hence, it is very essential to develop the PTT approach more safely and effectively.

## 3. Gold Nanostructures in Nanomedicine

Inorganic nanomaterials have more potential properties than organic nanomaterials due to colloidal stability, ease of synthesis in various adjustable sizes, and optical, magnetic, and quick surface modifications make them biocompatible materials in the area of biomedical applications [44,45]. Moreover, organic-based nanoparticles showed a higher degradation rate when compared to inorganic nanomaterials, this advantage makes them more colloidal stable in physiological conditions [46]. Among several types of inorganic nanoparticles, GNPs are promising candidates for photo therapies, due to their bioinertness, low cytotoxicity, as well as ease of preparation, and surface modifications [47]. Additionally, gold nanostructures are capable to improve the passive cellular uptake of phototherapies carriers in cancer sites through the enhanced permeability and retention (EPR) effect [48,49]. Further, GNPs hold a great surface area, which can aid in quick surface modifications with different bioactive molecules for active targeting in cancer therapy [50]. With the high binding affinity of GNPs with thiol and amine groups, the surface of GNPs can be modified with nucleic acids, proteins, and antibodies, which enable specific targeting ability and improve PSs delivery in tumor microenvironments [51]. The photostability of the GNPs leads to the high efficiency of absorbed laser radiation to thermal conversion which is photothermal conversion efficiency. The most important characteristics of GNPs is optical property originating from the localized surface plasmon resonance (LSPR) and huge surface-to-volume ratio that permits bioconjugation to bioactive molecules by a combination of various strategies of surface chemistry leads to develop conjugation of anticancer drug, targeting agent, and imaging probe in single nanoplatform [52]. In 1857, first time Faraday reported the synthesis of a colloidal solution of GNPs from the reduction of gold chloride (AuCl_4_) by phosphorus [44]. Turkevich and co-authors reported the colloidal GNPs through the chemical reduction of gold salt with trisodium citrate [53]. Currently, apart from spherical GNPs, various types of gold nanostructures of different morphology have been reported, such as nanoshells, nanostars, nanorods, and nanocages [54,55,56,57]. The LSPR indicates the coherent oscillation of excited electrons from the surface of the metals by absorbing strong NIR radiation [54]. The rise of this oscillation increased absorption of the electromagnetic radiation in the combination of SPR of the metallic nanomaterials, which is estimated by the morphology and size of the nanostructures.

The optical properties of metal nanomaterials have been extensively used in nanomedicine, from real-time molecular imaging to multimodal therapeutic based on the use of light, for instance, gaining from the efficient light to thermal conversion gold nanostructures [58]. One major issue that was raised on the usage of nanomaterials in clinical practice is the potential cytotoxicity of these nanoparticles. Many nanoparticles have been shown to indicate cytotoxicity for both cancer and healthy cells, because of oxidative stress and the stimulation of inflammatory effects [58,59,60,61]. These cytotoxicity effects are commonly based on the structure and size of the nanomaterials [62]. The GNPs with sizes ranging from 20–60 nm showed low cytotoxicity for biomedical applications. Their use has been progressively permitted by regulatory frameworks advanced by the Federal Food and Drug Administration [63,64]. Moreover, surface charge shows a strong influence on acute cytotoxicity, in GNPs with positive charges showed much more toxicity to tissues when compared to negatively charged nanomaterials [65,66]. Currently, GNPs are extensively applied in biomedical science, particularly in molecular diagnosis, biosensing, nanocarrier for drug delivery, and phototherapy agents owing to their electronic, optical, and colloidal stability. In presence of LSPR, GNPs may be suitable for therapy depending on the variety of wavelengths in the NIR, adapting them for nanomedicine as carriers in PDT or photothermal agents in cancer phototherapy. Indeed, when GNPs are treated by NIR laser radiation, most often in the NIR region (650–900 nm), they appear to be effective in converting photons to thermal waves. When GNPs are irradiated, they will scatter thermal waves in a localized path, generating an effective flow in temperature (40–42 °C), which sequentially has a deep influence on the survival of cancer cells that are lowly resistant to localized thermal waves when compared to healthy cells. From these results, hyperthermia has been suggested as a unique non-invasive cancer theranostics with localized thermal effects and without side effects to healthy tissues or cells. Many light sources have been suggested, from radiofrequency to laser are applied to stimulate the irradiation in gold nanostructures for phototherapy [67,68,69,70,71,72].

## 4. Optical Properties

### Effects of Size and Shape on Optical Properties of Gold Nanostructure

The optical properties of gold nanostructures differ from other metallic nanomaterials. When gold nanostructures are treated with light, the excited electrons from the GNPs surface generated coherent oscillations which are named LSPR [73]. Due to this LSPR, deep amplified and localized electromagnetic fields are produced in the gold nanostructure surface upon irradiation with a suitable wavelength of the laser. The absorbed NIR radiation by GNPs would decay radiatively by dispersing the emitting light with the same wavelength as the incident light, meanwhile the nonradiative relaxation, potentially the delivery of localized thermal energy. Hence, GNPs concentrate the orders of magnitude optical absorption, which in turn open the window for using the gold nanostructures as various bioimaging and biosensing agent beyond photo-associated therapies. As LSPR is influenced by the density and motion of electrons on the GNPs surface, the distinct size and variety of morphology of gold nanostructures depend on their scattering and plasmonic absorption. Gold nanostructures are commonly prepared through the reduction of gold salt using various reducing agents, and suitable reaction methodology has been widely applied to attain different sizes and shapes. Gold nanorods, nanoshells, nanocages, and nanorings have been widely studied as promising candidates for phototherapy. Based on biomedical applications, the NIR ranging from 750 to 1700 nm is better for tissue penetration because of low tissue scattering. Especially, the radiation ranging from 1000 to 1350 nm (second NIR window) can penetrate deepest than the range from 750 to 1000 nm (first NIR window). However, very low assessable biocompatible probes exist for pre-clinical applications in the NIR window. Incidentally, the probability of shifting the laser absorption and the plasmonic band of gold nanostructures from visible to NIR region by tuning their sizes and varying the shapes makes them hopeful replacements for in vivo applications in the phototherapy of cancer [21]. Currently, the appreciation of translation of LSPR within the suitable NIR region from anisotropic gold nanostructures has stimulated the preparation of a variety of gold nanostructures such as gold nanoclusters, nanostars, and nanoplates. Among many, certain common functional groups are associated with the gold nanostructures to attain favorable multi-functionality for cancer theranostics. Advanced nanotechnology area provides the chance to build GNPs of different sizes and morphology.

The physicochemical characteristics of GNPs differ among various functionalization and these characteristics could be accurately regulated through nanotechnology with the drive of connecting interdisciplinary fields (chemistry, biological, and physical) needs of the multifunctional phototherapies. Currently, five potential kinds of gold nanostructures have been widely investigated in the preclinical trials and they are hopeful for phototherapies: gold nanostructures (rods, shells, spheres, cages, and stars) are shown in Table 1.

Each of these gold nanostructures of various shapes holds certain special natures and their feature change openly depending on any rule. Important features of phototherapies, for instance, NIR absorbance and clearance rate, are majorly based on the shapes and sizes of these gold nanostructures. Materials science scientists must conduct various investigations to analyze the properties of these gold nanostructures to find those GNPs holds most desirable for phototherapies. Commonly, for a specific type of GNPs, the maximum extinction wavelength (λmax) increases with the increasing size of the gold nanostructures. As shown in Table 2. GNPs with smaller sizes exhibit much better absorption capacity and a minimum scattering-to-absorption efficacy ratio. Hence, the GNPs have larger size that exhibits higher scattering efficiency. In this regard, large-sized GNPs are recommended for good resolution and sensitive imaging-guided phototherapies in cancer and small-sized GNPs have higher absorption capability that is suggested for good photothermal conversion efficiency in phototherapies. As shown in Table 2. Determined maximum wavelength (λmax) and the ratio of scattering efficiency (µs) to absorption efficiency (µa) for GNPs with various shapes and sizes [74,75].

**Table 1:** The most common types of GNPs for efficient phototherapies application [75,76,77,78].

Types of Gold Nanostructures	Suitable Size of Phototherapies	Special Features
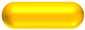 Nanorods	40–100 nm in length with an aspect ratio of 2–4	Nanorods have two SPR bands that might be used for biosensing applications.
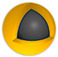 Nanoshells	100–150 nm diameter	Core@Shell
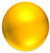 Nanospheres	5–100 nm	Easy to synthesize and bioconjugate with other bioactive molecules for therapeutic applications
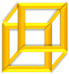 Nanocages	40–60 nm length	High drug loading capability
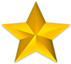 Nanostars	30–100 nm	Huge surface area

**Table 2:** Determined maximum wavelength (λmax) and the ratio of scattering efficiency (µ_s_) to absorption efficiency (µ_a_) for GNPs with various shapes and sizes [74,75].

Nanostructure Types	Dimensions (nm)	λmax (nm)	µ_a_ µm^−1^	µ_s_ µm^−1^	µ_s_/µ_a_
Silica@gold nanoshells	R_1_ = 50; R_2_ = 70	704	20.57	44.57	2.17
	R_1_ = 60; R_2_ = 70	892	35.66	22.73	0.64
	R_1_ = 120; R_2_ = 155	1160	7.26	15.44	2.13
Gold nanospheres	D = 20	521	73.72	0.45	0.01
	D = 80	549	67.88	45.94	0.68
Gold nanorods	R = 3.1; r_eff_ = 11.43	727	741.86	54.7	0.07
	R = 4.6; r_eff_ = 11.43	863	1003.37	102.05	0.1
	R =3.9; r_eff_ = 17.90	815	601.47	172.32	0.29

R—Aspect ratio; r_eff_—Effective radius.

## 5. Phototherapies-Based Clinical Trials in Gold Nanostructure

Over the past many eras, ongoing efforts have been made toward achieving an optimum association of nanoparticles and hyperthermia to overwhelm the issues of conventional thermal therapies. Among several reported nanoparticles for phototherapies applications, GNPs have been widely studied because of their high photothermal conversion efficiency and rapid surface modifications for improved targeted delivery of PS [79]. Plasmon resonance is a combined motion of a more number of mobile electrons [80,81,82]. In nanoparticle-mediated hyperthermia, GNPs can absorb strong NIR radiation and then produce the primary source of heat and reverse the direction of heat loss to stimulate thermal ablation on tumor cell [83,84,85,86]. Currently, various gold nanostructures have been translated for clinical trials (Table 3), potential struggle has resulted in the reports of multiple research works on gold-based phototherapies in the recent literature such as gold nanocages, nanobipyramids, nanoflowers, nanoshell, nanorods, hollow nanospheres, nanostars, nanoechius and plasmonic blackbodies [87,88,89,90,91,92,93,94,95,96,97,98,99,100,101]. However, significant translations of these nanomaterials to pre-clinical trials have not yet been attained. The potential clinical trial on phototherapies is mostly based on irradiation duration, gross irradiation dose, and mode of radiation delivery. Moreover, traditional passive targeting phototherapies based on gold nanostructures have been circumscribed in clinical trials because of their non-specific ability and generate side effects on normal tissues. Hence, currently designing active targeting GNPs-based phototherapies are under clinical trials.

## 6. PS Conjugated Gold Nanocarriers for PDT

Even though the PDT has significant efficiency for cancer therapy, still it’s mainly limited to in vivo investigations and loss-efficient translation into the clinical trial [110]. In this regard, nanomaterials conjugated PS-based PDT has developed as a modern approach in which nanoparticles act as carriers for PS gaining from the special properties that generate them as PS themselves [111]. Various chemotherapeutic agents have been delivered by functionalizing the surface of GNPs. Thus, GNPs have aided the specific-targeted delivery to cancer sites of different biomolecules utilized as therapeutic agents. PS-conjugated GNPs result in significantly increased electron transfer between the gold nanostructure and the photoactive dyes which improves the photodynamic efficiency [112]. Up to date only a few of these gold-conjugated PS-mediated PDT nanoplatforms achieved clinical trials. Recently, Anine Crous reported the application of AuNPs as a carrier for the AlPcS_4_Cl PS. In this study, synthesized gold nanoparticles were conjugated AlPcS_4_Cl, and in vitro models revealed that phototherapy using the AlPcS_4_Cl-gold nano bioconjugate was potential against human lung (A549) cancer cells [113]. In another approach, specific targeted PDT was carried out with folic acid (FA) and protoporphyrin IX (PPIX) conjugation on surface-modified AuNPs. The authors initially applied 6-mercapto-1-hexanol (MH) on the surface of AuNPs and it enhances the bioconjugation of FA/PPIX on nanoparticles to form a nanosystem (PPIX/FA-MH-AuNP). In this study, the synthesized nanosystem showed high cytotoxicity against HeLa cancer cells, due to the presence of targeting ligands FA and folate-mediated endocytosis. The results show that the PPIX/FA-MH-AuNP nanosystem increases the targeting nature and also phototoxicity efficiency of HeLa cells when compared with the traditional PDT [114]. In another approach, gold-based core@shell nanostructure was utilized to conjugate with PS for effective PDT. Ping and co-authors developed the nanosystem of Au@TiO_2_ nanostructure with hematoporphyrin monomethyl ether (HMME). The PDT efficiency of Au@TiO_2_-HMME was studied in KB cancer cells [115]. Dimakatso and co-authors fabricated the physically loaded hypericin (Hyp) on gold nanoparticles through sonication. The non-covalent bonds between the AuNPs and Hyp increase the PS accumulation in MCF-7 breast cancer cells and hence improve PDT efficiency with the minimum concentrations. From this study, it is revealed that physically loaded Hyp PS on AuNPs is a hopeful strategy for hydrophobic PS drug delivery to improve PDT efficiency [116]. The recent studies involving PS-conjugated AuNPs for PDT against cancer using in vitro models are listed in Table 4.

**Figure 1:**
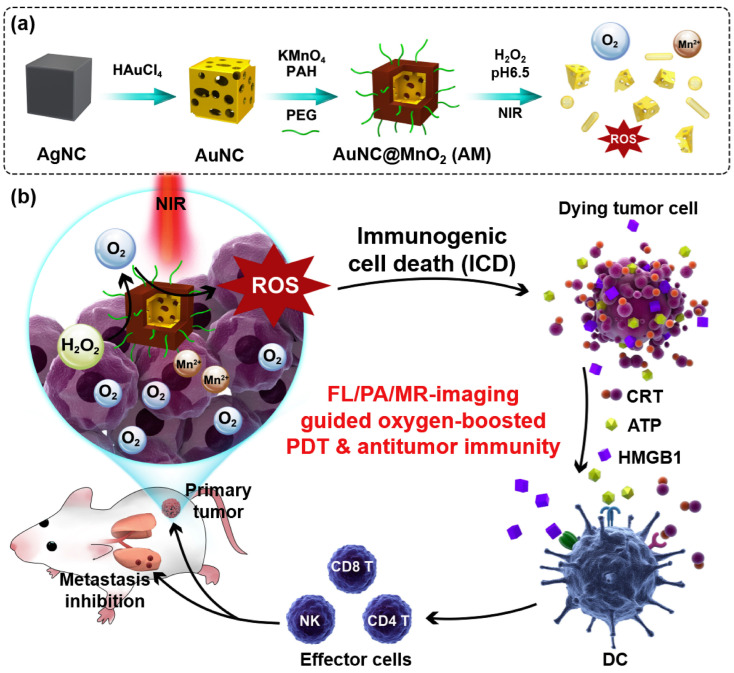
Schematic representation of (**a**) synthesis of AuNC@MnO_2_ (AM) and (**b**) imaging-guided PDT ef-ficiency of AuNC@MnO_2_ (AM) reproduced with permission [125]. Copyright 2018 Elsevier.

In another approach, Liand and coworkers developed novel nanocage-based core@shell gold@manganese dioxide (Au@MnO_2_) nanoparticles to produce oxygen to PDT against breast cancer shown in Figure 1. Under NIR irradiation, self-producing oxygen from gold nanocages improved the PDT efficiency, on the other hand stimulating immunogenic cell death (ICD) through damage-associated molecular patterns [125]. Similarly, Dai and coworkers fabricated the g-C_3_N_4_ nanosheets functionalized with gold (gC_3_N_4_-Au NPs). In this study, below 670 light treatments on gC_3_N_4_-Au NPs, the results showed that nanoparticles can produce more ROS and destroy tumors [121]. Thus, attention should be considered in the selection of PDT drugs, carriers, and the mode of administration of the PSs inside the body to prolong their circulation in the body to obtain an improved prognosis [32].

## 7. Gold Nanostructures-Based Targeting Hypoxia for PDT

**Figure 2:**
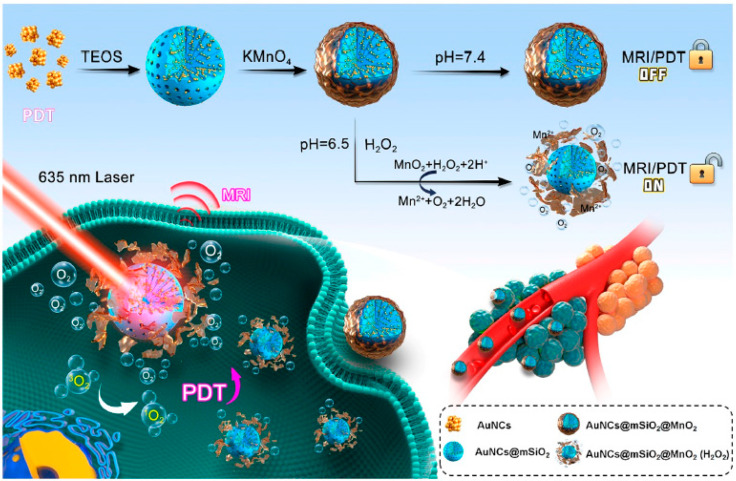
Schematic representation of synthesis and PDT efficiency of AuNC@mSiO_2_@ MnO_2_ (H_2_O_2_) nanosystem. Reproduced with permission [131]. Copyright 2021 ACS.

Hypoxia commonly exists in the tumor microenvironment due to the excess proliferation of cancer cells that surpass the blood circulation and oxygen supply for the growth of weak vasculature tumors. It has been revealed that hypoxic efficiently promotes cancer development and invasion [126]. The potentiality of PDT is restricted by hypoxia since the traditional PDT is oxygen dependent to generate reactive oxygen species (ROS). Hence, targeting hypoxia in the tumor sites in absence of oxygen-based PDT has developed as a potential tool to enhance PDT. A recent review by Lou-Franco reported that gold nanostructures hold efficient enzymatic activities for instance reductase, peroxidase, oxidase, and superoxide dismutase, which can be leveraged to relieve hypoxia [127]. In a recent study, an oxygen self-producing nanosystem containing metal-organic frameworks, AuNPs and Ce6 PS was utilized to diminish the hypoxic which consequently improves PDT. In this approach, AuNPs are applied to catalase efficiently by catalyzing the high H_2_O_2_ developed in the tumor sites to generate oxygen molecules to reduce hypoxia and improve ROS generation with high cytotoxicity [128]. Moreover, to reduce hypoxia and improve PDT, core@shell Au@Rh nanoparticles based on Au and rhodium (Rh) with catalase activities were applied as nanoenzyme. The ICG was conjugated with Au@Rh and functionalized with a cancer cell membrane (CM) to develop Au@Rh-ICG-CM nanosystem. The nanosystem catalyzed the H_2_O_2_ in the tumor sites to form oxygen via the action of Au@Rh to improve ROS generation for efficient PDT. The functionalization with CM permits specific targeting via homology adsorption [129]. In a similar approach to rise the oxygen range in the hypoxia, Au@MnO_2_ core@shell nanoparticles based on Au and MnO_2_ (manganese dioxide) were formulated for oxygen-improving immunogenic PDT shown in Figure 2. The nanosystem responds to lower pH at the tumor site and is also assisted as the tumor stimulates oxygen production upon light irradiation to generate ROS. In the presence of hypoxia, the slow degradation of the MnO_2_ shell in the lower pH of the tumor sites consequently produces oxygen, thereby improving the PDT efficiency through light irradiation. In this study, the nanosystem diminishes hypoxia at the tumor site and stimulates immunogenic ablation of cancer kills [130].

In a very recent study, Yin and co-authors reported gold nanoclusters (AuNCs), mesoporous silica (mSiO_2_), and MnO_2_-based nanoenzyme system with oxygen generators for improved PDT and magnetic resonance imaging. In this study, the nanosystem made up of AuNCs doped on mSiO_2_ was coated with MnO_2_ and acted as a shell (AuNCs@mSiO_2_@MnO_2_). In lower pH, becomes turn ‘on’ the nanoenzyme is treated with H_2_O_2_ consequently resulting in the degradation of MnO_2_. The slow degradation of MnO_2_ generates massive oxygen molecules, which in turn enhances Magnetic Resonance Imaging and PDT [131].

## 8. Synergetic Phototherapies Based on Gold Nanostructures

The boundaries of cancer nanotheranostics are constantly being altered as better thought of biomedical characteristics of nanomaterials is expanding. Over the last two decades, there is an outstanding evolution in the fabrication and advantages of various nanostructures in cancer nanotheranostics [132]. In this regard, GNPs have currently emerged as the most hopeful theranostic agent [133,134]. The gold nanostructures are the potential ability for passive accumulation and preferentially reach the tumor microenvironment through enhanced permeability and retention effect of the infected organs [135]. In addition to that, the surface of gold nanostructures can be readily modified with biomolecules such as peptides, monoclonal antibodies, and proteins to overcome the non-specific targeting ability of theranostics agents [136,137,138,139]. Due to their natural biointerness, surface area-to-volume ratio, and surface chemistry, gold nanostructures are widely applied as nanocarriers for targeted drug delivery [140]. The high atomic numbers in gold are capable to afford a higher X-ray absorption cross-section, making them promising nanomaterials to act as potential radiosensitizers for improving radiotherapy (RT) [141]. Moreover, the photothermal conversion efficiency of gold nanostructures can be used to produce localized thermal for tumor ablation [142]. In recent years, the thermal efficiency of gold nanomaterials has been discovered and utilized to help hyperthermia in curing cancer. According to this, the radiofrequency (RF) electrical field absorption efficiency of gold nanostructure combined with their sonosensitizing characteristics has been employed to enhance the RF and ultrasound-stimulated hyperthermia therapeutic efficiency [143]. In addition to these, the thermal efficiency of gold nanostructures can be applied to stimulate responsive drug payload delivery with good specific and resolution upon external triggering through hyperthermia materials [144,145]. Currently, many biomaterials scientists have also revealed that gold nanostructures are capable to produce cytotoxic ROS upon laser and ultrasound treatments, and could therefore be applied to promote PDT and sonodynamic therapy simultaneously [146,147]. With these characteristics combined into a single nanoplatform, gold nanostructures have emerged as an entirely irreplaceable nanostructure ability to combine various therapies to attain improved therapeutic outcomes. Hence, they could deliver a golden choice to combine various therapies in a single mode, thus emphasizing the influence of gold nanostructure in synergetic cancer theranostics. Taking the benefit of gold nanostructures for real-time co-delivery of various therapies cannot only improve the potentiality of every individual cancer treatment but fascinatingly may also deliver an extra advantage by the synergistic interactions that happen among different therapies, which results in very powerful therapeutic outcomes. This synergistic therapy also aids to limits high dose concentrations and associated side effects by permitting a reduction in administered drugs level for cancer treatments [148]. However, a lot of synergetic therapy modals have been reported for the gold nanostructures, the upcoming section only focuses on phototherapies-based synergetic therapies using various gold nanostructures especially; the combination of photodynamic therapy with others will be discussed. Table 5, provides a summary of the therapeutic aids of various gold nanostructures for synergetic phototherapies for cancer theranostics.

### 8.1. Synergetic PDT-CT-PTT

In recent years, traditional single-modal cancer treatment has become hopeless because of the complex formulation and alterable tumor microenvironment of growing cancer. For instance, Chemotherapy (CT) is the main treatment method for curing cancer, but this method suffers from poor biological barriers, non-specific nature, and drug resistance, and causes heavy injury to the healthy cells in the clinic [168]. Therefore, establishing combined therapeutic approaches based on various therapy modalities retains considerable efficiency in the area of both basic and clinical research. Several investigations have shown that nanoparticle-based synergetic therapy is a very hopeful approach to overwhelm the above-mentioned restrictions due to its combined therapeutic efficiency and low drug dosage [169,170,171,172]. Currently, the combination of CT, PDT, and PTT is creating more attention because of the outstanding sustainable drug release, improved tumor ablation, and low cytotoxicity [168]. The remarkable therapeutic abilities of synergetic PDT-PTT, [173,174,175] CT-PDT, [176,177] and CT-PTT [178,179] are also established. Even though several double-modality therapy nanocarriers have been extensively well-organized, the nanostructure-dependent synergetic triple-modality PDT/PTT/CT therapy remains in the beginning stage. Furthermore, this combined therapy is vital to enhance therapeutic efficiency, low drug dosage, and minimum laser radiation for phototherapy [180]. Recently, certain multifunctional nanocarrier systems based on carbon nanotubes [181], MnO_2_ [182] and upconversion nanocarriers [183] have been developed for multi-modal PDT-CT-PTT treatments.

**Figure 3:**
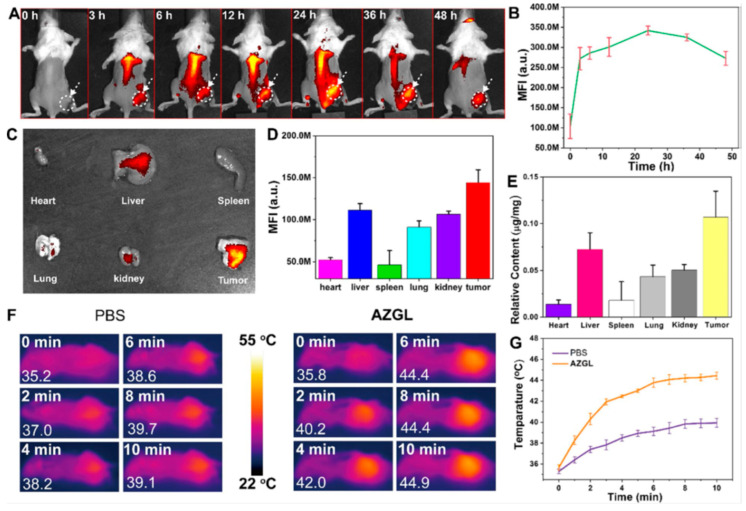
(**A**) In Vivo FL images (**B**) mean fluorescence curve after i.v. of AZGL (**C**) Ex-vivo fluorescence imaging of organs (**D**) mean fluorescence intensity of tumor and organs after i.v. of AZGL (**E**) The relative content of Au treated mice after i.v. of AZGL. (**F**) In Vivo IR thermal imaging and (**G**) the temperature change curves after i.v. of PBS and AZGL with 980 nm laser treatment for 10 min. Reproduced with permission [184]. Copyright 2022 Springer Nature.

In recent years, several multimodal therapies based on gold nanostructure have been reported. Among them, phototherapies combined with chemotherapies have great interest due to the surface plasmon resonance property of gold nanostructure. In this regard, recently Li and co-authors developed a multifunctional nanosystem with gold-based core@shell nanostructure that was fabricated as a single nanoplatform for the synergetic PDT-PTT-CT against breast cancer. In this work, initially, the gold nanostars (AuNS) were functionalized with gambogic acid (GA) and Zr^4+^ with tetrakis (4-carboxyphenyl) porphyrin (TCPP) to form the nanosystem as AuNS@ZrTCPP-GA (AZG). The functionalized gold nanostars were coated with PEGylated liposome (LP) to generate core@shell AuNS@ZrTCPP-GA@LP (AZGL) nanosystem. At low pH of the tumor microenvironment, the resultant nanosystem under slow degradation of liposomes the core materials of AuNS, GA, TCPP, and Zr^4+^ was released to perform the synergistic therapy towards cancer through the combination of AuNS-based PTT and TCPP-based PDT. The entire GA acts to decrease the thermal resistance of the cancer cells to improve PTT and the developed nanoplatform exhibited outstanding therapeutic efficiency in vivo model as shown in Figure 3. The fabricated AuNS@ZrTCPP-GA@LP (AZGL) nanosystem demonstrated a pH-responsive therapeutic efficiency for the potential gold nanostructure-based synergistic treatment of breast cancer (Figure 3) [184].

In another system, Xu and co-authors designed a PEG functionalized gold nanorods mediated nanosystem with pH-dependent drug release character for triple-modality PDT-PTT-CT treatment against cancer. In this approach, the surface of GNRS was functionalized with mercapto propionyl hydrazide (MPH) and thiolated PEG which consequently linked with therapeutic agents doxorubicin (DOX) and PS 5-aminoevulinic acid (ALA) through hydrazine bonds. The resulting nanosystem GNRs-MPH-ALA/DOX-PEG demonstrated good stability in normal physiological pH and unstable in acidic pH due to the presence of hydrazone bonds between drug and MPH conjugated gold nanostructures. In this study, in vitro investigations exhibited that nanosystem could potentially be uptake by MCF-7 cells and release the payload of DOX and ALA into the nucleus. On laser irradiation, PS generated ROS for PDT, on the other hand, GNRs could potentially stimulate thermal efficiency for PTT. Compared with mono or dual therapies, the triple modal CT-PDT-PTT therapies could more potentially destroy cancer cells through super-additive anticancer effects [157]. Thus, the combination of CT, PDT, and PTT is creating more attention because of the outstanding sustainable drug release, improved tumor ablation, and low cytotoxicity.

### 8.2. Synergistic PDT-PTT

To improve antitumor efficiency and precise therapy, the combination of multimodal therapeutic approaches leading to synergistic effects are successful approach [185,186]. Co-assembly of various therapeutic agents has received significant research attention in cancer therapy [186,187,188,189,190]. Among all the cancer therapies PDT-PTT involving laser radiation has unique benefits, including minimal cytotoxicity, non-invasive, remote controllability, and low side effects [191]. The NIR photo-therapeutic window for cancer ranges from 700 to 1100 nm, where the absorption of the laser radiation by soft tissues and blood is low, permitting curing cancers through thermal ablation. For PDT, NIR-stimulated PSs can produce ROS, such as free radicals, peroxides, and singlet oxygen, which can destroy the tumor cells [192,193,194]. The combination of various nanostructure with photosensitizers provides the dual PDT-PTT in a single nanoplatform and improve the therapeutic efficiency for cancer. The gold nanostructure can perform photothermal agents in PTT and nanocarrier for photosensitizers in PDT, hence gold nanostructure-based phototherapies are promising synergistic therapies in cancer treatments. Recently, Sun and coworkers fabricated a novel gold nanostructure “nanodendrite” by standardizing the geometrical configurations, further gold nanodendrite (AuND) was functionalized with mitochondria targeting molecules (triphenyl phosphonium, TPP), and photosensitizers (indocyanine green, ICG), further coated with the macrophage cell membrane (MCM) for encapsulation of AuND, TPP, and ICG to form the nanosystem (AuND-TPP-ICG@MCM). The novel nanosystem can carry out multimodal imaging (fluorescence-photoacoustic-surface enhanced Raman imaging) and therapy in NIR-II (PTT) and NIR I (PDT) for cancer therapy. Improved hyperthermia and higher generation of ROS in the tumor microenvironment through MCM functionalization and mitochondria targeting provide a synergistic efficiency for tumor ablation with low side effects.

In this approach, the nanosystem exhibited biocompatibility, multimodal imaging, and efficient therapeutic conditions indicating the significance of this nanosystem for clinical practice in cancer treatments [195]. In another approach, Li and co-workers, designed the gadolinium ions covered gold nanoparticles (ultra-small size) form, similar to the core@shell type nanosystem. Further, gold nanostructures were functionalized with matrix metalloproteinase-2 (MMP-2) and pay loaded with IR820 for synergistic therapies (PTT-PDT) for liver cancer. In this approach, the prepared nanosystem was metabolized in vivo through biodegradation under bimodal imaging due to its acidic stimulated degradation nature. Moreover, the nano-materials exhibited outstanding specific targeting capability due to the presence of MMP-2. In vivo investigations showed that the nanomaterials attainted improved synergistic PDT-PTT therapies under laser treatments and effectively reduced tumor growth. From this study, it concludes that the gold nanostructure-based nanosystem has great efficiency against liver cancer with a dual imaging system [196]. Synergistic phototherapies have been shown to encourage therapeutic efficiency against various types of cancers. Designing a delivery approach for specific targeting and deep-penetrating tumors is still the main challenge for advancing these synergistic phototherapies. Chanung and co-authors designed a novel method by using stem cells for the delivery of photodynamic and photothermal agents for cancer treatments. In this approach, gold nanorod (AuNRs) functionalized PEG, PEI, and Chlorin e6 (Ce6), further loaded with stem cell (ADSC). In this system, AuNRs act as photothermal agents, while Ce6 for PDT. The photothermal treatment was stimulated by applying NIR light irradiation at 808 nm encouraged the release of Ce6 from the stem cells into the tumor microenvironment [159]. Although Among combinational therapies, PDT-PTT provides multiple benefits, including minimal cytotoxicity, non-invasive, remote controllability, and low side effects, however, there is a need for clinical studies to evaluate the post-treatment complications.

### 8.3. Synergistic PDT-RT

Radiation therapy (RT) is a class of extensively applied methods for cancer therapy in clinical practice. The therapeutic efficiency of RT is significant because it destroys tumor regions with no depth limitation using high energy [197]. Several approaches have been applied to reduce the dose level of X-rays to decrease the side effects of x-ray radiation [198]. However traditional radiosensitizers lack specific targeting ability for tumors [199,200]. Currently, several high atomic number nanomaterials have been developed such as gold nanostructures, MoS_2_/Bi_2_S_3_, and CuS nano materials perform as radiosensitizers [201,202]. Due to the specific tumor-targeting ability, strong NIR absorption, and deposition of energy from photo-electrons, the above-mentioned nanomaterials can improve the RT efficiency of the cancer region and minimum side effects to healthy cells, resulting in the improvement of mean survival of case studies [203,204]. Hence, synergistic radiotherapy with chemotherapy is often applied in clinical practice. In PDT, most of the clinically applied organic photosensitizers can be stimulated by visible light, for surface tumors only [205]. Compared to organic photosensitizers, metal-mediated therapeutic agents have high molar extinction coefficients, good photostability, and minimum enzymatic degradation rate [206]. Noble metal-based gold, silver, and platinum nanomaterials have been showing the generation of singlet oxygen with the treatment of visible light [207]. Hence, it is essential to develop a NIR-absorbing PDT agent for deep-tissue tumor therapy. Recently, gold nanocages have been established that it can generate ROS under laser irradiation [208]. In addition, gold nanocages can act as radiosensitizers for improved radiotherapy [209]. Hence, the synergistic therapies of PDT-RT would potentially enhance its therapeutic efficiency [210,211]. The gold nanostructures can absorb strong NIR radiation, which can stimulate thermal efficiency for PTT and thermoelastic expansion for photoacoustic imaging [212,213,214,215]. It is important to note that hyperthermia holds dangerous side effects for the radio-resistant cells of the S-phase, consequently improving cell sensitivity.

### 8.4. Synergetic PDT-IT

In the last decade, cell-mediated delivery nanocarriers for cancer therapy have become a novel research interest in the area of nanomedicine. Various types of stem cells have been reported for specific targeting abilities to cancer cells because of their property of cancer homing. The developed immune cells loaded with therapeutic agents can potentially pass into the tumor microenvironment through the blood vessels, and attain combined therapeutic efficiency [216,217]. Currently, gold nanostructures-based theranostics applications had reached a peak in the area of cancer imaging, PTT, and PDT [218]. In recent years, multiple research work has been reported based on the combination of immune therapy with other therapies using various nanoparticles for cancer theranostics. In this regard, Fangfang and co-authors reported the gold nanostructure-based synergistic immune therapy (IT) and PDT for cancer treatment. In this approach, gold nanoclusters were self-assembled with Ce6 molecules to form Ce6-GNCs. Further, the nanostructure was labeled with CD3 antibody and CIK cells to generate a CIK cell-mediated nanosystem (Ce6-GNCs-Ab-CIK). The nanosystem showed good specific targeting ability and therapeutic efficiency against MGC-803 tumor-bearing mice. This approach revealed that the synergistic therapeutic effect of GNCs-Ce6-Ab-CIK exhibits the combination of PDT and IT therapy [155]. In the human body, the immune system consists of NK cells, these cells plays important role in the prevention of the formation and growth of cancer cells. NK cells can identify and remove the cancer cells which were independent antigens and antibodies [219]. In addition to that no requirement of grafting host disease due to the absence of T cell receptors in the NK cell surface [220]. The immune response-based NK cells are majorly by the release of various kinds of cytokines, which take part in a potential role in the research area of synergistic therapies in cancer [221,222]. In this regard, Liu and co-authors designed a novel nanosystem based on NK cells with gold nanostructure for synergistic therapies of PDT-PTT-IT. In this approach, the gold nanostars (GNSs) loaded with Ce6 molecules and NK cells, further encapsulated with CaCO_3_ to form GNS@CaCO_3_/Ce6-NK nanoplatform. In this approach, the prepared nanoplatform exhibited good specific targeting ability toward A549 cancer-bearing mice models [165]. This novel platform provides a synergistic approach for improved photodynamic, photothermal, immunotherapy, radiotherapy, and chemotherapy in the war against cancer soon.

## 9. Major Challenges in Using Gold Nanostructures for Medical Applications

Currently, there are various golden standard approaches for testing the cytotoxicity of nanostructure in vitro. Moreover, biomedical scientists have designed suggestions for finding the acute/chronic cytotoxicity of several nanoparticles [223]. For various cancer cells, the effect of gold nanostructure occurs at various amounts of concentrations. In this regard, it can be established that it is essential to generate various universal procedures that subsequently assess the biosafety of gold nanostructure in each particular case study, which will be applied to all same nanostructures throughout the world [224,225] The development of a novel model or structure that would change the surface could aid in naturally exhibiting the characteristics of nanostructure by their physicochemical properties [226]. Even though investigations on gold nanostructure are very appropriate, still not been comprehensive analysis on their renal clearance, release kinetics, and bioavailability in the physiological system. There is a less accurate number of investigations on the pharmacokinetics of gold nanostructures at the physiological condition, which limits the probability of the large use of gold nanomaterials in curing cancer diseases. It is significant to note that gold nanostructures are flexible materials for investigating inside the organism, compared with various other nanomaterials, due to their good extinction coefficient and localized surface plasma resonance [227]. An investigation reported by Wilhelm S and co-authors revealed the nanoparticles proceed to cancer sites and observed that, on average, only 0.7% of nanoparticles achieved the cancer sites. Moreover, when nanoparticles are administrated in vivo, the phagocytic system and the renal clearance pathway shallow almost all nanoparticles, consequently reducing therapeutic efficiency and severely injuring the phagocytic system [228]. Currently, there are some clinical trials applying gold nanostructures (https://clinicaltrials.gov accessed on 15 January 2023), which do not yet permit future investigations on several factors such as clearance, biodistribution, and protein sorption. Hence limit the applications of gold nanomaterials in the medical field. The beginning of clinical trials could establish and understand the therapeutic and imaging importance of nanoparticles [229].

## 10. Conclusions and Outlook

Over many decades, abundant novel designs of gold nanostructures with good optical properties have been established. Even though outstanding trust and advancement in plasmonic gold nanostructures in nanomedicine applications, still there is a large gap between basic research and clinical translations for the gold nanostructure. For cancer-based applications, now it is beginning the synergistic therapies use gold nanostructure and takes duration to achieve the clinical trials. The combination of phototherapies with other traditional therapies allows for minimizing toxic doses and retaining the therapeutic potential efficiency. It is expected that the applications of gold nanostructures for phototherapies will progressively be transformed into the clinics they deliver the controlled action. When combined, gold nanostructures may transport not only various therapeutic agents to achieve the specific cancer region but also localize the thermal waves at particular locations, which would standardize clinical producers and be regulated by the clinician. The synergistic therapeutic approaches are capable to decrease the side effects of a mono-modal therapy, resulting in a better quality of life.

## Figures and Tables

**Table 3:** Clinical trials on GNPs for phototherapies and other cancer-related health issues.

Clinical Trial ID	Product Name	Gold Nanostructures	Pathology	Therapies	Ref.
NCT01270139	NANOM FIM	Silica-gold nanoparticle	Atherosclerotic lesions	Plasmonic photothermal therapy	[102]
NCT01436123	NANOM PCI	Gold nanoparticles with silica-iron oxide shells	Atherosclerosis	Plasmonic photothermal and stem cell therapy	[103]
NCT00848042	AuroLaseR	Silica-gold nanoshells coated with PEG	Head and neck cancer	Thermal ablation of solid tumors via NIR laser	[104]
NCT03020017	NU-0129	A spherical nucleic acid (SNA) gold nanoparticle	Gliosarcoma, glioblastoma	Safety evaluation of NU-0129	[105]
NCT02755870	CNM-Au8	Nanocrystal of gold	Healthy volunteers	tolerability of CNM-Au8	[106]
NCT01420588	AuNPs	Gold nanoparticles	benign gastric lesions	Gastric lesions detection	[107]
NCT02782026	AuNPs	Gold nanoparticles	heritable PAH	Detection of pulmonary arterial hypertension (PAH)	[108]
NCT00436410	CYT-6091 (Aurimune)	TNF-bound colloidal Gold	metastatic cancer	Selective tumor trafficking	[109]

**Table 4:** Most recent studies involving PS conjugated AuNPs for PDT against cancer using in vitro models.

Gold Nanostructures	Nanosystem	Photosensitizers	In Vitro Cell Studies	Ref.
AuNPs	AlPcS_4_Cl-AuNPs	AlPcS_4_Cl	A549	[113]
AuNPs	PPIX/FA-MH-AuNP	PPIX	HeLa	[114]
Au@TiO_2_	Au@TiO_2_-HMME	HMME	KB	[115]
Au NPs	Au-Hyp	Hyp	MCF-7	[116]
AuNPs	Au-AlPcS_4_Cl	AlPcS_4_Cl	A375	[117]
AuNPs	Au-(OH)2P(V) (py)Ga(III) A3 type meso-triarylcorroles	A3 type meso-triarylcorroles	MCF-7	[118]
Au nanocage	PTX-PP@Au NPs	PP	PC-3 cancer cells	[119]
Au nanostars	GNS@BSA/I-MMP2 NPs	MMP2	NA	[120]
AuNPs	g-C_3_N_4_-AuNPs	g-C_3_N_4_	A549, MCF-7 and HeLa cells	[121]
Au Nanorods	AuNR@SiO2-TCPP	TCPP	A549 cells	[122]
Au Nanorods	ICG loaded Au@SiO_2_@mSiO_2_	ICG	HepG2 cells	[123]
Au nanocage	GC-pep@SiNC-AuNC	SiNC	U87 cells	[124]

**Table 5:** The summary of the therapeutic aids of various gold nanostructures for synergetic phototherapies for cancer theranostics.

Gold Nanostructures	Multifunctional Nano Platforms	Therapeutic Agents	Imaging Model	Therapy Model	In Vitro Models	Ref.
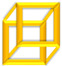 **Nanocages**	BPQD-AuNCs	DOX and QUR	FL	PDT-CT	MCF-7/ADR	[149]
	DOX/ICG-biotin-PEG- AuNC-PCM	DOX and ICG	FL	PDT-CT-PTT	MCF-7/ADR	[150]
	AuNCs-HA	HA	PA	PDT-RT-CT	4T1	[151]
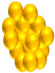 **Nanocluster**	Ce_6_-GNCs-DOX	Ce6 and DOX	FL	PDT-CT	A549	[152]
	Ce_6_-GNCs-Ab-CIK	Ce6 and CD3 antibody	FL	PDT-IT	MGC-803	[153]
	AuS-U11	5-ALA and Cy5.5	FL	PDT-PTT	PANC1-CTSE	[154]
	Gd_2_O_3_-AuNCs-ICG	ICG	MRI/CT	PDT-PTT	HeLa	[155]
	Au NCs-INPs	ICG	NIRF/PA	PDT-PTT	4T1	[156]
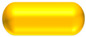 **Nanorods**	GNRs-MPH-ALA/DOX-PEG	5-ALA and DOX	NIRF/PA	PDT-CT-PTT	MCF-7	[157]
	FA-PEG-P(Asp)- DHLA-AuNR100-SS-Ce_6_	Ce6	-	PDT-PTT	MCF7 and A549	[158]
	AuNR-PEG-PEI-APP/Ce_6_—ADSC	Ce6	-	PDT-PTT	MCF-7	[159]
	AuNRs-Ce_6_-MSNRs	Ce6	NIRF/PA	PDT-PTT	4T1	[160]
	PEG-GNR-ACPI	PpIX	FL	PDT-PTT	SCC-7	[161]
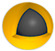 **Nanoshells**	GGS-ICG	ICG		PDT-PTT	4T1	[162]
	Pt@UiO-66-NH2-Aushell-Ce_6_	Ce6	FL	PDT-PTT	MCF-7	[163]
	ICG-Au@BSA-Gd	ICG	NIRF/PA/CT/MR	PDT-PTT	4T1	[164]
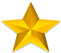 **Nanostars**	GNS@CaCO3/Ce6-NK	Ce6	NIRF/PA	PDT-IT-PTT	A549	[165]
	GNS-PEG-Ce6	Ce6	FL	PDT-PTT	A549	[102]
	GNS@BSA/I-MMP2	MMP2	NIRF/PA	PDT-PTT	A549	[166]
	GNS@CaCO3/ICG	ICG	NIRF/PA	PDT-PTT	MGC803	[167]

## Data Availability

Not applicable.
